# Acute Type A Aortic Dissection During Vascular Endothelial Growth Factor Tyrosine Kinase Inhibitor Lenvatinib Therapy

**DOI:** 10.1155/crcc/3082479

**Published:** 2025-10-07

**Authors:** Jo-Ju Wu, Yen-Chun Hsu

**Affiliations:** ^1^ Department of Nursing, National Taiwan University Cancer Center, Taipei, Taiwan; ^2^ Department of Anesthesiology, National Taiwan University Cancer Center, Taipei, Taiwan

## Abstract

A 67‐year‐old male with hepatocellular carcinoma under lenvatinib therapy suffered from initially back pain and then abdominal pain, dyspnea, and oliguria. Acute type A aortic dissection was diagnosed with point‐of‐care ultrasound and computed tomography during the admission. Patients treated with vascular endothelial growth factor tyrosine kinase inhibitors (VEGF‐TKIs) have a low but significant risk of aortic dissection. As a newer member of VEGF‐TKIs, lenvatinib has a similar or even higher risk of aortic dissection compared to others. Clinicians should take the cardiovascular risk into consideration while prescribing lenvatinib and keep the differential diagnosis of aortic dissection in mind during the therapy.

## 1. Introduction

Vascular endothelial growth factor (VEGF) tyrosine kinase inhibitors (TKIs) (VEGF‐TKIs) have been the mainstream therapy for patients with various advanced malignancies. However, aortic dissection is a rare but potentially fatal complication in cancer patients treated with VEGF‐TKI therapy. Lenvatinib, a relatively new VEGF‐TKI, has been approved by the US Food and Drug Administration (FDA) since 2015 for several malignant cancers. To date, no case report of lenvatinib‐associated aortic dissection has been published. We report a case of aortic dissection during lenvatinib therapy and discuss the association and risk of aortic dissection in patients receiving this treatment.

## 2. Case

A 67‐year‐old male with no prior history of hypertension or other major systemic diseases except hepatitis B was diagnosed with hepatocellular carcinoma (HCC) in 2015. He had undergone surgical intervention and transcatheter arterial chemoembolization in 2015 and 2021, respectively. In July 2022, he underwent a left liver lobectomy due to recurrence of HCC. Lenvatinib was initiated in August 2022 as adjuvant therapy following the surgery. Prior to starting lenvatinib, echocardiography revealed mild aortic root dilation (4.4 cm) and contrast‐enhanced chest computed tomography showed no aortic atherosclerosis. After starting lenvatinib, the patient developed hypertension and hand–foot syndrome. The hypertension was initially managed with amlodipine (5 mg daily) and later supplemented with valsartan (40 mg daily).

In mid‐September 2022, he began experiencing back pain. Whole‐body bone scintigraphy, performed to assess for bone metastasis, showed no significant findings. On October 2, 2022, he went to the emergency room and then was admitted with new‐onset abdominal pain, dysuria, and dyspnea. Initial vital signs were as follows: temperature 36.6°C, pulse rate 109/min, respiratory rate 21/min, and blood pressure 143/88 mmHg. A chest X‐ray revealed bilateral lung infiltrates. Pneumonia and lenvatinib‐induced interstitial pneumonitis were considered in the differential diagnosis. Empirical treatment with piperacillin/tazobactam and methylprednisolone (1 mg/kg/day) was initiated.

**Figure 1:**
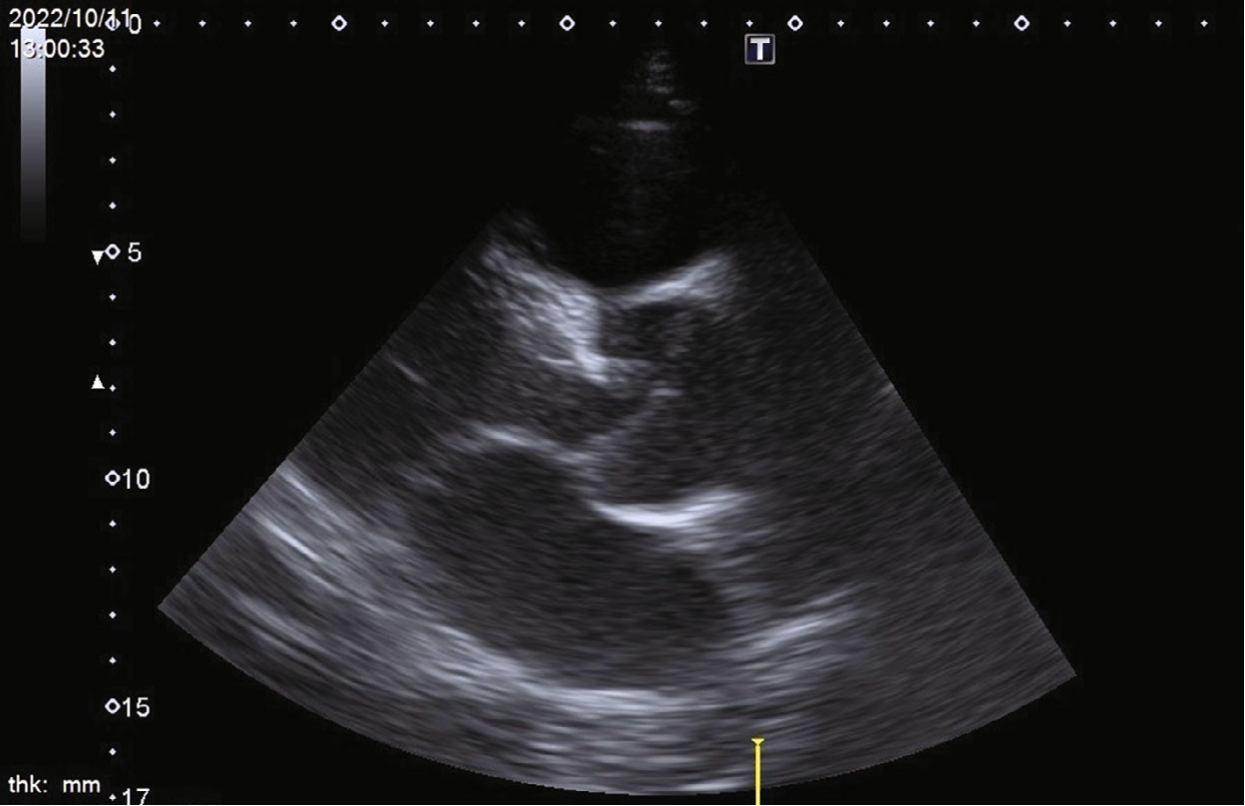
POCUS image: a fluttering flap at the aortic root.

**Figure 3:**
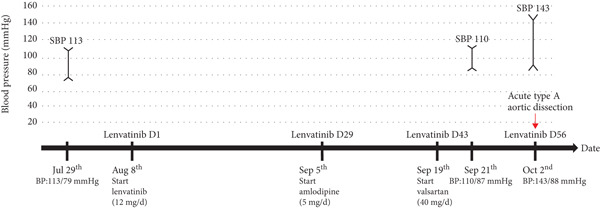
Timeline of events and blood pressure trend.

His abdominal pain was alleviated with acetaminophen/tramadol. Over the next week, he developed weight gain (from 79 to 93 kg), lower leg edema, and oliguria with acute kidney injury (creatinine increased from 1.4 to 2.9 mg/dL). Progressive dyspnea ensued, and on October 9, 2022, he was transferred to the intensive care unit. Continuous renal replacement therapy was initiated for acute kidney injury with fluid overload. The following day, a chest X‐ray showed cardiomegaly and bilateral pleural effusion. Point‐of‐care ultrasonography (POCUS) revealed a dilated inferior vena cava with no respiratory variation, left ventricular global hypokinesia with an ejection fraction of 30%, and a fluttering intimal flap at the aortic root with moderate aortic regurgitation (Figure 1). Acute type A aortic dissection was confirmed by contrast‐enhanced computed tomography (Figure 2). Laboratory findings included CK 103 U/L, CK‐MB 4.73 U/L, troponin T 92.50 ng/L, and NT‐proBNP > 35,000 pg/mL. He was transferred immediately by ambulance to National Taiwan University Hospital on October 11, 2022. On the same day, he underwent ascending aortic grafting with left common carotid artery and descending aorta endovascular stenting, as well as coronary artery bypass grafting to the right coronary artery. The postoperative course was smooth. He was discharged on November 16, 2022, without complications (Figure 3).

Figure 2(a) CT image, transverse plane: intimal tear at the aortic root (arrow). (b) CT image, coronal plane: intimal tears at the aortic root and ascending aorta (arrowheads).

**Figure:**
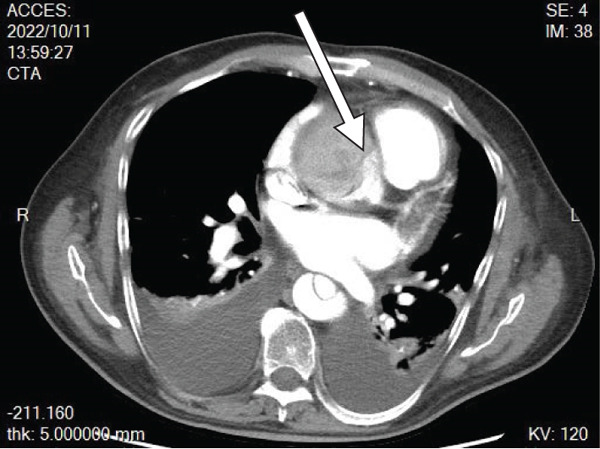
(a)

**Figure:**
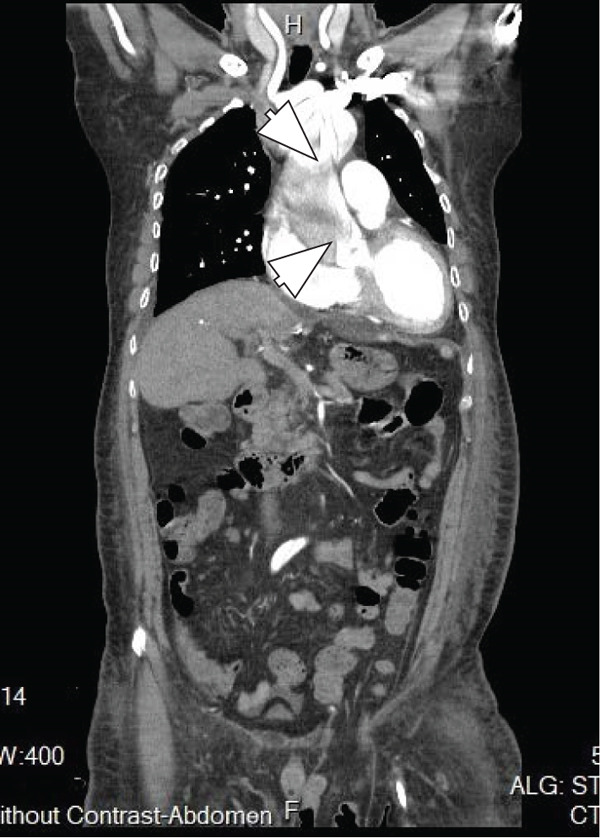
(b)

## 3. Discussion

This case highlights the occurrence of aortic dissection during lenvatinib therapy. Although statistical analyses have identified aortic dissection as an adverse event associated with VEGF‐TKIs, including lenvatinib, no case reports of lenvatinib‐associated aortic dissection have been published. We present this case to document and explore the correlation with the risk of aortic dissection during lenvatinib therapy.

Lenvatinib, a multireceptor TKI, was first approved by the US FDA in 2015 for the treatment of radioiodine‐refractory differentiated thyroid cancer. Later, it was also approved for the treatment of advanced renal cell carcinoma, advanced endometrial carcinoma, and unresectable HCC [1]. The main mechanism of lenvatinib is targeting VEGF receptors, fibroblast growth factor (FGF) receptors, platelet‐derived growth factor receptor alpha (PDGFR*α*), KIT, and RET [2]. Since VEGF and FGF signaling is critical to angiogenesis, a process essential for tumor growth and metastasis, inhibiting these pathways slows angiogenesis and tumor progression. Additionally, inhibiting VEGF and FGF signaling may exert direct antitumor effects through immunomodulatory mechanisms [3]. In HCC, lenvatinib is approved as a first‐line therapy for unresectable or advanced disease in patients without prior treatment in the United States, EU, Japan, and China, based on the results of the Phase III REFLECT trial [4]. In this trial, lenvatinib demonstrated noninferiority to sorafenib in terms of overall survival, with significant improvements in objective response rate, progression‐free survival, and time to progression [5]. The most common adverse events associated with lenvatinib in this study were hypertension (42%), diarrhea (39%), decreased appetite (34%), and weight loss (31%).

However, aortic dissection is a rare but potentially fatal adverse event associated with VEGF‐TKIs. The two main mechanisms underlying VEGF‐TKI‐associated aortic dissection are thought to involve interference with the integrity of the aortic wall and hypertension. First, VEGF is widely distributed throughout the body and plays a key role in the vasculature of nontumor tissues. By inhibiting VEGF in both tumor and nontumor tissues, VEGF‐TKIs may increase vascular stiffness, interfere endothelium regeneration, and weaken the media layer of the aortic wall, predisposing it to dissection [6]. Second, VEGF‐TKIs induce hypertension. Several mechanisms have been proposed for VEGF‐TKI‐induced hypertension, including activation of the endothelin system, reduced nitric oxide bioavailability, stimulation of renin–angiotensin–aldosterone system, and microvascular rarefaction [7–9]. Notably, the rapid onset of hypertension following VEGF‐TKI administration, and its prompt resolution upon drug withdrawal, suggests that those functional alternations in vascular tone are likely primary contributors to the hypertensive response [7]. Hypertension is a common side effect of most VEGF‐TKIs [7, 10]. A meta‐analysis of 72 randomized controlled trials involving 30,013 patients showed a 23% incidence of all‐grade hypertension associated with VEGF‐TKIs and a 4.4% incidence of high‐grade hypertension [10]. The time to onset of hypertension is typically short. In some early clinical studies, elevated blood pressure was observed within 24 h of initiating VEGF‐TKI therapy [11, 12]. The combination of the two factors may synergistically increase the risk of aortic dissection.

Although aortic dissection associated with VEGF‐TKIs is rare, case reports and retrospective analyses using adverse drug event databases have shown a significant correlation. An analysis using Japanese Adverse Drug Event Report (JADER) database extracted spontaneous adverse drug reaction reports between 2004 and 2015 [13]. Of the 16,441 patients treated with systemic VEGF‐TKIs, 59 (0.065%) had aortic dissection. The patients treated with VEGF‐TKIs had a significant high crude odds ratio, which is 22.3 (95% CI: 11.2–49.4), compared to the reference group of cancer patients who were not treated with VEGF‐TKIs. Another review of cases reported to US FDA Adverse Event Reporting System (FAERS) between 2004 and 2019 also supports the association between VEGF‐TKIs and arterial aneurysm and dissection [14]. In the review, which includes 240 arterial aneurysm/dissection cases, 22% of cases were reported to have fatal outcomes related to the event.

It remains unclear whether lenvatinib carries a higher risk of aortic dissection than other VEGF‐TKIs. The JADER database showed one case of aortic dissection in 96 lenvatinib‐treated patients, with a crude odds ratio of 78.5 (1.8–566.3), the highest among all VEGF‐TKIs [13]. Similarly, a study using the FAERS database identified 31 lenvatinib‐associated aneurysm and dissection cases among 634 cases involving all VEGF‐TKIs [15]. The reporting odds ratio for lenvatinib‐associated aneurysms and dissections was 3 (2.11–4.28), which was higher than most other VEGF‐TKIs (Table 1). In addition, patients treated with lenvatinib also appear to have a higher incidence of hypertension, which may predispose aortic dissection, compared to those receiving other VEGF‐TKIs [5, 16, 17]. A recent meta‐analysis reports that the relative risk for all‐grade and high‐grade hypertension in patients receiving lenvatinib was 2.61 and 3.35, respectively (both *p* ≤ 0.01), compared with other VEGF‐TKIs or placebo [16]. However, due to the small number of cases, it is still difficult to conclude whether lenvatinib carries a higher risk of aortic dissection than other VEGF‐TKIs.

**Table 1:** Comparison of published studies from JADER and FAERS databases.

**Data source**	**Author**	**Study period**	**No. of all VEGF-TKI-related cases**	**No. of lenvatinib-related cases**	**Odds ratio of AD/AAD of lenvatinib users**	**Conclusion**
JADER database	Oshima, Y. et al. [13]	2004–2015	49 cases of AD among 16,441 patients	1 case of AD among 96 patients	Crude odds ratio 78.5 (95% CI: 1.8–566.3)	Highest odds ratio among all VEGF‐TKIs
FAERS database	Wang, S. et al. [15]	2004–2020	634 cases of AAD among 196,559 patients	31 cases of AAD among 6392 patients	Reporting odds ratio 3 (95% 2‐sided CI: 2.11–4.28)	Lower odds ratio than that of ramucirumab, ranibizumab, and bevacizumab, yet higher than that of other VEGF‐TKIs

Abbreviations: AAD, aneurysm and artery dissection; AD, aortic dissection; FARES, US FDA Adverse Event Reporting System; JADER, Japanese Adverse Drug Event Report.

We report this case of a previously normotensive patient who developed acute type A aortic dissection within 55 days of receiving lenvatinib therapy. While causality cannot be definitely established, the presence of mild aortic root dilation may have been a contributing factor. Moreover, the clinical presentation of our case is relatively vague. It took us some time to make the decision to perform image studies and diagnose the acute aortic dissection. According to previous reports, the median time to onset of arterial aneurysms and dissection in lenvatinib‐treated patients is 82 days (range: 1–371 days), compared to 94 days (range: 1–1955 days) in patients treated with other VEGF‐TKIs [14]. However, the onset time varies significantly among different VEGF inhibitors, ranging from 13.5 days for regorafenib to 494.5 days for ponatinib. Approximately 10.87% of lenvatinib‐associated arterial aneurysms and dissections occur within 120 days, which is relatively rapid compared to other VEGF‐TKIs [15].

In conclusion, lenvatinib, like other VEGF‐TKIs, carries a small but notable risk of aortic dissection, which can be fatal. It is essential to carefully evaluate the risks and benefits of lenvatinib therapy, particularly in cancer patients with pre‐existing cardiovascular risk factors. Routine home blood pressure monitoring immediately after starting VEGF‐TKI therapy and early initiation of antihypertensive treatment may help mitigate the risk of VEGF‐TKI‐associated aortic dissection. Whether lenvatinib presents a higher risk than other VEGF‐TKIs warrants further investigation. Clinicians should maintain a high level of suspicion for aortic dissection in patients receiving lenvatinib or other VEGF‐TKIs and manage such cases promptly and appropriately.

## Consent

The patient allowed personal data processing, and informed consent was obtained from his legal representative. The patient was informed that identifying information would be removed to ensure anonymity.

## Conflicts of Interest

The authors declare no conflicts of interest.

## Funding

No funding was received for this manuscript.

## Data Availability

The data that support the findings of this study are available from the corresponding author upon reasonable request.
